# Treatment of pathological fractures due to brown tumours in a patient with hyperparathyroidism and lack of parafibromin expression – A case report

**DOI:** 10.1016/j.tcr.2020.100367

**Published:** 2020-10-14

**Authors:** Michał Wasiak, Michał Popow, Magdalena Bogdańska, Aleksandra Starzyńska-Kubicka, Paweł Małdyk, Piotr Wasilewski

**Affiliations:** a Department of Orthopaedics and Traumatology, Medical University of Warsaw, Lindleya 4, 02-005 Warsaw, Poland; b Department of Internal Diseases and Endocrinology, Medical University of Warsaw, Banacha 1a, 02-097 Warsaw, Poland; c Department of Pathology, Medical University of Warsaw, Pawińskiego 7, 02-106 Warsaw, Poland

**Keywords:** Brown tumour, Femur fracture, Hypercalcemia, CDC73, Parafibromin

## Abstract

Brown tumours, known also as osteitis fibrosa cystica, are benign osteolytic lesions found in 5–15% of patients with hyperparathyroidism, and commonly located in mandibles, the shafts of long bones, the pelvis or ribs. As they compromise bone strength, pathological fractures can be a typical effect of their presence; but given the complex nature of the disease process in this case, such fractures require an interdisciplinary approach directed at orthopaedic treatment, plus management of the underlying hyperparathyroidism. In this paper, we present the case of a 36-year-old female patient with bilateral anophthalmia, hyperparathyroidism and nephrolithiasis, in whom a fall led to her sustaining a pathological fracture of the proximal third of the femoral shaft in the place of an osteolytic lesion, as well as second pathological fracture of the left patella also changed by multiple examples of such lesions. Parathyroidectomy on account of adenoma had been performed 2 weeks prior to the trauma. The femoral shaft fracture was treated surgically, the patella fracture conservatively, and a sample brown tumour was found in tissue. As the parathyroid showed no parafibromin expression, a diagnosis of HPT-JT (hyperparathyroidism and jaw tumour) was arrived at, with this condition given as caused by *CDC73* mutation. This disease is able to account for brown tumours, hyperparathyroidism, benign or malignant tumours of kidneys, intestinal tract, and lungs. The approach combining treatment of the fractures with intervention over the parathyroid adenoma proved a successful one, with complete bone union ensuing, and no relapse into hyperparathyroidism 2 years on from the surgery. This case indicates the importance of an interdisciplinary approach to the treatment of brown tumours, as well as the necessity for a diagnosis to be extended when incidental brown tumours are found.

## Introduction

Brown tumours, known as osteitis fibrosa cystica, are benign osteolytic lesions of bones caused by excessive functioning of osteoclasts and hence the resorption of the mineral component from bony tissue [1,2]. Under microscopic examination they present a developed form of osteitis fibrosa cystica making it necessary for giant cell tumour to be precluded [1]. A characteristic feature is the replacement of bone marrow and cancellous bone by hypervasculated connective tissue [1]. The most common locations for lesions of this kind are the mandibles, the shafts of long bones, the pelvis or ribs. The prevalence of brown tumours in patients with primary hyperparathyroidism is 5%, though rising to even 15% in some regions. The cause of onset of the abovementioned processes remains unclear, but is probably hyperthyroidism accompanied by other predisposing factors like end-stage renal disease or genetic disorders [1,2,4].

The presence of brown tumours impairs bone strength to the point where sufferers are predisposed to pathological fractures. Treatment of these proves a difficult clinical task, given that adequate reduction and stabilisation must go hand in hand with management of hyperparathyroidism.

Hyperparathyroidism and jaw tumour syndrome (HPT-JT) can be an underlying condition in patients with brown tumours. It is a genetic disease, caused by *CDC73* mutation, characterised by impaired parafibromin production in tissues. It may lead to parathyroid cancer as well as benign and malignant tumours of kidneys, intestinal tract, lungs and endometrium.

## Case presentation

A 36-year-old woman with bilateral anophthalmia and hyperparathyroidism in the course of a parathyroid adenoma and nephrolithiasis complicated by renal failure (G-3) with GFR 53 ml/min/1.73 m2, was admitted to an Orthopaedic Department on account of fractures to the shaft of the right femur and the left patella being the result of a fall. Surgical parathyroidectomy had been performed 2 weeks prior to trauma.

The patient was in a good state overall, though reporting severe pain in the right hip that prevented active movement, mild swelling of the thigh, and swelling of the left knee with pain in the left patella on palpation. There was no compromising of blood-flow, sensation or motor-nerve function.

**Fig. 1:**
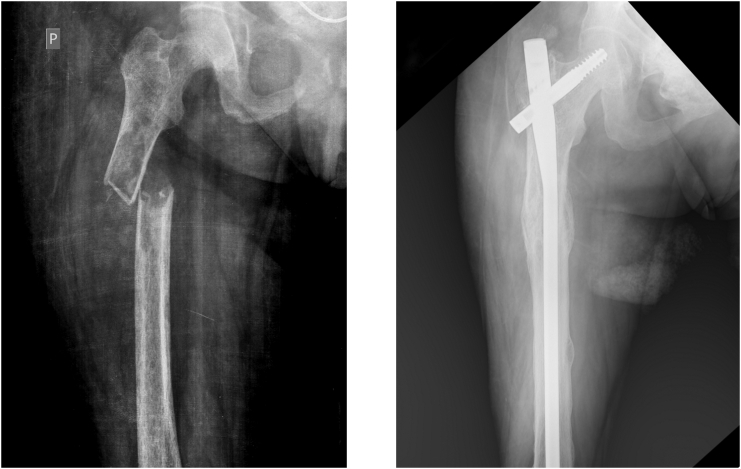
Pathologic fracture of the right femur (on left) and one year after surgical treatment (on right).

**Fig. 2:**
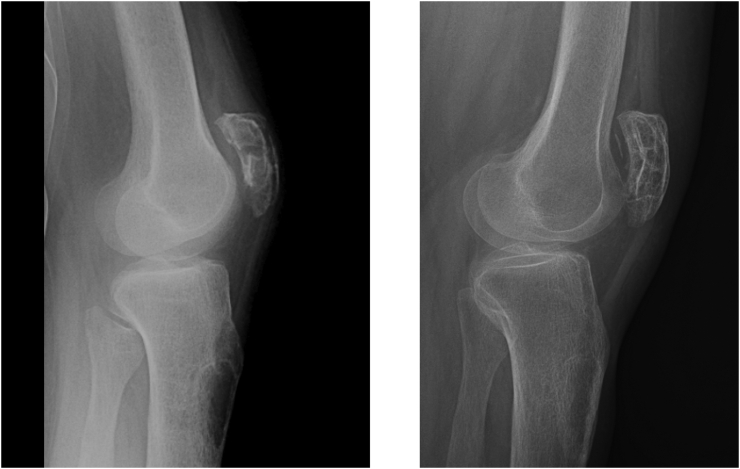
Pathologic fracture of the left patella (on left) and one year after conservative treatment (on right).

Under X-ray a pathological fracture of the proximal third of the femoral shaft was revealed, in the place of an osteolytic lesion of diameter 60 mm, with thinning of the cortical layer (Fig. 1). In turn, the second pathological fracture involved the left patella visibly changed by multiple osteolytic lesions (Fig. 2). Further multiple osteolytic lesions were in fact found on the left tibia and the left pubic and sciatic bone.

Given the history of parathyroid adenoma, disseminated brown tumours were suspected. The patient was scheduled for surgical treatment of the femoral shaft fracture, and, given the lack of displacement and the extensive bone osteolysis, also for conservative treatment of the patella fracture. An inferior vena cava filter was implanted prior to surgery, given a history of inferior vena cava and bilateral common iliac vein thrombosis.

The procedure performed was closed reduction and internal fixation (CRIF) with a proximal femoral nail. A tissue sample was obtained for histopathology, the results sustaining the initial diagnosis of pathological fracture reflecting the presence of a brown tumour.

As the surgery was not followed by further complications, the patient was discharged home in a good overall state. And, as she did not attend for initial after-surgery checks, the first such check of an orthopaedic nature was performed one year on from the operation. A CT-scan and X-Rays then showed full bone union at both fracture sites, with no secondary displacement, and with brown-tumour regression seen to be taking place in the lower limbs and pelvis (Fig. 1, Fig. 2). The patient reported no pain and was able to walk with the assistance of another person. She is now checked regularly by an endocrinologist, and, 2 years after parathyroidectomy, there is no sign of a relapse into hyperparathyroidism.

## Discussion

The term osteitis fibrosa cystica, which also encompasses brown tumours, is taken to refer to osteolytic bone lesions caused by hyperparathyroidism, given the excessive activation of osteoclasts. The process causes cortical bone to thin and become cancellous, with the marrow portion in retreat, its place taken by hypervasculated and mechanically insufficient connective tissue [1]. The lesion does not form metastases, and in most cases recedes following normalisation of parathyroid hormone levels [3]. In the meantime, the compromising of bone strength predisposes sufferers to pathological fractures. Brown tumours occurrence is notably high among women with hyperparathyroidism in their 30s and 40s [2]. Histologically, these take the form of giant cell tumours [1].

In the differential diagnosis of any osteolytic bone lesions, it is particularly important to pay attention to calcium and phosphorus homeostasis [6,7,9]. The clinician should raise a suspicion of brown tumour when abnormalities in their levels accompany high concentration of parathyroid hormone. When such bony lesions are located in the mandible, as it was a case in our patient, it is strongly suggestive and requires diagnostics for hyperparathyroidism and jaw tumour syndrome (HPT-JT) [7].

HPT-JT is caused by a mutation of the CDC73 gene, which encodes parafibromin [8]. Parafibromin is a protein regulating metabolic pathways of NOTCH, Hedgehog, Wnt and polymerase, which are of crucial importance to the most of the cell functions. The abnormal structure of the parafibromin molecule may promote uncontrolled cell proliferation and lead to carcinogenesis. Lack of its normal expression in parathyroid glands confirmed a diagnosis of HPT-JT in our patient (sFig. 3). HPT-JT increases the risk of parathyroid cancer, as well as benign and malignant tumours of the kidneys, intestinal tract, lungs and endometrium [5,10]. This knowledge is important from the point of view of further endocrinological, internal and gynecological diagnostics. In the described patient a benign tumour of the uterus was also found.

**Fig. 3:**
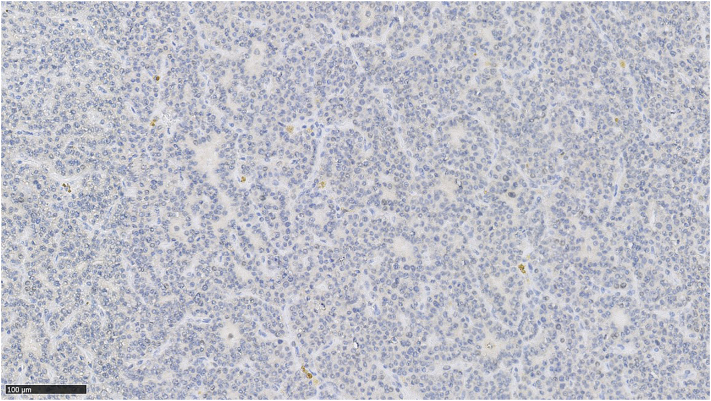
Negative staining for parafibromin in the parathyroid gland.

Standards for prophylactics via-a-vis brown-tumour-related pathological fractures have still not been set, but lowering of parathyroid hormone levels seems the most important intervention if such fractures are to be averted. Such normalisation of PTH – achieved by parathyroidectomy – leads to spontaneous healing [1,4]. In the meanwhile, in active individuals with brown tumours located in their lower limbs, preventive intramedullary nailing can be performed. Mirels Criteria can prove helpful in the relevant decision-making process.

Proper treatment of brown tumour-related pathological fractures requires a multidisciplinary approach, focused on the achievement of appropriate bone union following on from the treatment of the underlying disease, as well as orthopaedic management. The principles of orthopaedic treatment do not differ from the widely-accepted methods of managing long-bone shaft fractures, being based on stable osteosynthesis with preservation of proper length, axis and rotation of the bone. Intramedullary nailing remains the preferred stabilisation technique, with time to bone union seen to depend on how well under control the underlying hyperparathyroidism may be.

Brown tumours found incidentally prove to be an absolute indication when it comes to diagnosing disorders of the metabolism of calcium and phosphate. Some of these may have a genetic background, and hence require screening for accompanying diseases.

## Declaration of competing interest

All authors declared no conflict of interest.
